# Morphological and molecular characteristics of a *Trypanosoma* sp. from triatomines (*Triatoma rubrofasciata*) in China

**DOI:** 10.1186/s13071-024-06274-w

**Published:** 2024-05-10

**Authors:** Yunliang Shi, DeHua Lai, Dengyu Liu, Liyan Du, Yuanyuan Li, Xiaoyin Fu, Peichao Deng, Lili Tang, Shanshan He, Xiaoquan Liu, Yanwen Li, Qin Liu

**Affiliations:** 1 Parasitology Department, School of Basic Medical Sciences, Guangxi Medical University, Nanning, 530021 People’s Republic of China; 2 Guangdong Provincial Key Laboratory of Aquatic Economic Animals, State Key Laboratory of Biocontrol, School of Life Sciences, Sun Yat-Sen University, Guangzhou, 510275 People’s Republic of China; 3 National Institute of Parasitic Diseases, Chinese Center for Disease Control and Prevention (Chinese Center for Tropical Diseases Research); National Key Laboratory of Intelligent Tracking and Forecasting for Infectious Diseases; Key Laboratory on Parasite and Vector Biology, Ministry of Health; WHO Collaborating Centre for Tropical Diseases; National Center for International Research on Tropical Diseases, Ministry of Science and Technology, Shanghai, 200025 People’s Republic of China; 4 Key Laboratory of Basic Research on Regional Diseases (Guangxi Medical University), Education Department of Guangxi Zhuang Autonomous Region, Nanning, 530021 People’s Republic of China

**Keywords:** *Trypanosoma* sp., *Triatoma rubrofasciata*, Morphology, Molecular characteristics, *Trypanosoma conorhini*, Infection prevalence

## Abstract

**Background:**

Triatomines (kissing bugs) are natural vectors of trypanosomes, which are single-celled parasitic protozoans, such as *Trypanosoma cruzi*, *T*. *conorhini* and *T*. *rangeli*. The understanding of the transmission cycle of *T*. *conorhini* and *Triatoma rubrofasciata* in China is not fully known.

**Methods:**

The parasites in the faeces and intestinal contents of the *Tr*. *rubrofasciata* were collected, and morphology indices were measured under a microscope to determine the species. DNA was extracted from the samples, and fragments of 18S rRNA, heat shock protein 70 (HSP70) and glycosomal glyceraldehyde-3-phosphate dehydrogenase (gGAPDH) were amplified and sequenced. The obtained sequences were then identified using the BLAST search engine, followed by several phylogenetic analyses. Finally, laboratory infections were conducted to test whether *Tr*. *rubrofasciata* transmit the parasite to rats (or mice) through bites. Moreover, 135 *Tr*. *rubrofasciata* samples were collected from the Guangxi region and were used in assays to investigate the prevalence of trypanosome infection.

**Results:**

*Trypanosoma* sp. were found in the faeces and intestinal contents of *Tr*. *rubrofasciata*, which were collected in the Guangxi region of southern China and mostly exhibited characteristics typical of epimastigotes, such as the presence of a nucleus, a free flagellum and a kinetoplast. The body length ranged from 6.3 to 33.9 µm, the flagellum length ranged from 8.7 to 29.8 µm, the nucleus index was 0.6 and the kinetoplast length was −4.6. BLAST analysis revealed that the 18S rRNA, HSP70 and gGAPDH sequences of *Trypanosoma* sp. exhibited the highest degree of similarity with those of *T*. *conorhini* (99.7%, 99.0% and 99.0%, respectively) and formed a well-supported clade close to *T*. *conorhini* and *T*. *vespertilionis* but were distinct from those of *T*. *rangeli* and *T*. *cruzi*. Laboratory experiments revealed that both rats and mice developed low parasitaemia after inoculation with *Trypanosoma* sp. and laboratory-fed *Tr*. *rubrofasciata* became infected after feeding on trypanosome-positive rats and mice. However, the infected *Tr*. *rubrofasciata* did not transmit *Trypanosoma* sp. to their offspring. Moreover, our investigation revealed a high prevalence of *Trypanosoma* sp. infection in *Tr*. *rubrofasciata*, with up to 36.3% of specimens tested in the field being infected.

**Conclusions:**

Our study is the first to provide a solid record of *T*. *conorhini* from *Tr*. *rubrofasciata* in China with morphological and molecular evidence. This Chinese *T*. *conorhini* is unlikely to have spread through transovarial transmission in *Tr*. *rubrofasciata*, but instead, it is more likely that the parasite is transmitted between *Tr*. *rubrofasciata* and mice (or rats). However, there was a high prevalence of *T*. *conorhini* in the *Tr*. *rubrofasciata* from our collection sites and numerous human cases of *Tr*. *rubrofasciata* bites were recorded. Moreover, whether these *T*. *conorhini* strains are pathogenic to humans has not been investigated.

**Graphical Abstract:**

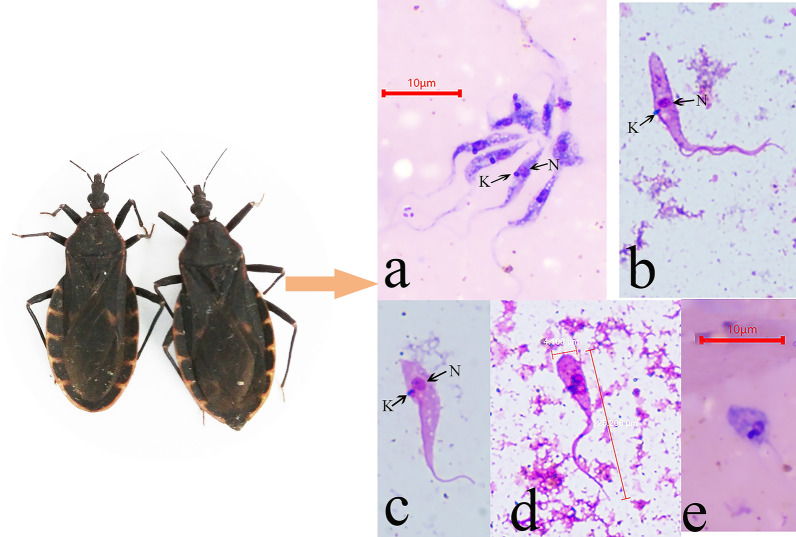

**Supplementary Information:**

The online version contains supplementary material available at 10.1186/s13071-024-06274-w.

## Background

The *Trypanosoma* genus comprises single-celled protozoan parasites. They are composed of seven subgenera, three of which are stercorarians and four of which are salivarians [1, 2]. These parasites are widespread globally and can infect both humans and diverse animal species [3]. They are blood-borne parasites and can be transmitted by haematophagous vectors, such as leeches, biting flies and kissing bugs [4]. These parasites cause diseases that are both economically and socially significant, such as sleeping sickness in equatorial Africa and Chagas disease in Latin America [5]. *Trypanosoma brucei* is transmitted by tsetse flies and causes sleeping sickness. There are two forms of this disease: a chronic form caused by *T*. *b*. *gambiense* infection in West and Central Africa and an acute form caused by *T*. *b*. *rhodesiense* in East Africa [6, 7]. Similarly, *T*. *cruzi* is mostly transmitted by insects in the subfamily Triatominae and causes Chagas disease, which is a significant human disease causing approximately 6 million infections in the Americas [8]. Chagas disease can lead to both high morbidity and mortality rates among adults in endemic countries, leading to more than 10,000 deaths annually [8].

 Triatominae insects, commonly called “kissing bugs”, may spread many medically important microorganisms and viruses, especially pathogens, such as *T*. *cruzi*, which may threaten public health [9]. In addition to harbouring *T*. *cruzi* infection, triatomines can carry many *Trypanosoma* species, such as *T*. *rangeli* and *T*. *conorhini*. The two latter species have not been associated with symptomatic human pathologies or diseases [10]. However, nonhuman primates are commonly infected with these parasites, and the geographical distribution of *T*. *rangeli* often overlaps with that of *T*. *cruzi*, as they often share the same vertebrate and invertebrate hosts. This overlap between nonmedically and medically important species could lead to incorrect diagnosis of *T*. *cruzi* infections on the basis of morphological examination [11]. *Trypanosoma conorhini* was reported to have a mysterious host range in rats, mice and nonhuman primates [12, 13]. In many Asian countries, *Tr*. *rubrofasciata* has been reported to be infected with *T*. *conorhini* [14, 15], with countries, such as Vietnam, having reports of coinfections with both *T*. *conorhini* and *T*. *lewisi* [15]*.* However, the current data available about the distribution of *Trypanosoma* species in Asia have mostly been based on morphological identification methods. Therefore, there is currently insufficient molecular evidence that *Tr*. *rubrofasciata* carries *T*. *conorhini* in China.

In Asia, there are currently seven species of *Triatoma*, of which only two species (*Tr*. *rubrofasciata* and *Tr*. *sinica*) are commonly found in China [16]. The presence of *Tr*. *rubrofasciata* in southern China has recently drawn the attention of the public because it tends to bite humans [17, 18]. In 2023, two new *Triatoma* species (*Tr*. *picta* and *Tr*. *atrata*) were recognized [19]. Previous studies revealed that *Tr*. *rubrofasciata* is widely distributed in southern China (e.g. in Guangdong Province, Hainan Province and the Guangxi Zhuang Autonomous Region), where people are reportedly bitten [20, 21]. In this study, flagellates were found in field-captured *Tr*. *rubrofasciata*. We were therefore interested in documenting the prevalence of *Trypanosoma* infections in South China populations; *Tr*. *rubrofasciata* was used to investigate the morphological and molecular characteristics of these trypanosomes and to analyse which animal species they can infect.

## Methods

### Triatomine sample collection for parasite identification

Triatomines were collected from piles of wood in Chongzuo City and Beihai City from June to November 2021–2022 in the Guangxi Zhuang Autonomous Region, including Jiangzhou district (22.40520083N, 107.35192533E) and Hepu County (21.8213004237N, 109.4140264191E). The samples were transferred to the laboratory for feeding. The species of the collected triatomines were identified using both morphological and molecular biology methods as previously described [18, 21].

### Parasite morphology identification

Trypanosomes were observed in triatomines by diluting the faeces and intestinal contents with phosphate-buffered saline (PBS), smearing the solution onto a microscope slide and observing the slides under a microscope. Moreover, the same sample smearing after Giemsa staining were observed. Similarly, trypanosomes in rat/mouse blood samples treated with PBS and Giemsa stain were observed under a microscope. Identification was performed on the basis of the parasites’ morphological features as described by Hoare [3]. Briefly, after taking the photo, centre-to-centre distances were measured using ImageJ v1.54 [22], i.e. from the posterior end to the nucleus (PN), from the posterior end to the kinetoplast (PK), from the kinetoplast to the nucleus (KN) and from the anterior end to the nucleus (NA); body length (BL), not counting the free flagellum, i.e. posterior end to the anterior end; free flagellum length (FF); total length (TL), i.e. BL + FF; cell maximum body width (BW); nucleus width (NW); and nucleus length (NL). The indices representing the position of the nucleus (NI = PN/NA), kinetoplast (KI = PN/KN) and flagellum (FI = FF/BL) were also calculated.

### Parasite molecular identification

#### DNA extraction and polymerase chain reaction

Using the QIAamp DNA Mini Kit (Qiagen, Germany), DNA from 200 µl of gut content from each triatomine was extracted according to the manufacturer’s recommendations. The 18S rRNA, heat shock protein 70 (HSP70), glyceraldehyde-3-phosphate dehydrogenase (gGAPDH) and internal transcribed spacer region (ITS) gene sequences were amplified via PCR (the primer information for each gene is listed in Table 1). The reactions were conducted in a final volume of 25 μl, consisting of 30 ng of DNA template, 1 × Taq PCR Master Mix (Takara, China), and 0.4 μM of each primer. The fragments were amplified with the following thermal cycling conditions: 95 °C for 3 min; 35 cycles of 94 °C for 30 s, 48–58 °C for 60 s, and 72 °C for 90 s; and 72 °C for 10 min.

**Table 1:** The primer information

Target gene	Sequence 5′-3′	Annealing temperature	Amplicon size	References
18 s rRNA	ZC18-f:5-CATATGCTTGTTTCAAGGAC-3 ZC-18-r:5-GACTTTTGCTTCCTCTATTG-3	45 °C	~ 2200 bp	[43]
HSP70	H70F:5-TGATGCAGCTGGTGTCGGACTT-3 H70R:5-CTGGTACATCTTCGTCATGATG-3	58 °C	~ 800 bp	[30]
GAPDH	GAF:5-GGBCGCATGGTSTTCCAG-3 GAR:5-CCCCACTCGTTRTCRTACC-3	55 °C	~ 800 bp	[29]
ITS	TRYP1S:5-GGAAGCCAAGTCATCCATCG-3 TRYP1R:5-CGTCCCTGCCATTTGTACACAC-3	55 °C	652 bp	[44]
Nest PCR (18 s rRNA)	TRY816F CAGAAACGAAACACGGGAG TRY816R CCTACTGGGCAGCTTGGA	55 °C	~ 900 bp	[24]
	SSU450F-TGGGATAACAAAGGAGCA SSU450R-CTGAGACTGTAACCTCAAAGC	55 °C	~ 600 bp	

The amplicons were purified using the MiniBEST Agarose Gel DNA Extraction Kit (Takara, Dalian, China) according to the manufacturer’s recommendations and subsequently conjugated with the PMD20-T vector (Takara, Dalian, China). Two positive clones were sent to Sangon Biotech (Shanghai, China) for sequencing of both strands. The sequencing reactions were performed using the ABI Prism® BigDye® Terminator v3.1 Cycle Sequencing Kit (Applied Biosystems) with RimersM13 in an ABI 3730 sequencer.

#### Molecular analysis and phylogenetic analysis

The obtained 18S rRNA, HSP70, gGAPDH and ITS sequences were compared with those available in the GenBank database (NCBI) by the Basic Local Alignment Search Tool (BLAST) (http://blast.ncbi.nlm.nih.gov/Blast.cgi) to identify the homologous sequences and download sequences from different trypanosome species. Multiple sequence alignments of homologous trypanosomes, such as *T*. *conorhini*, *T*. *vespertilionis*, *T*. *cruzi*, *T*. *rangeli*, *T*. *wauwu*, *T*. *dionisii*, *T*. *erneyi* and *T*. *livingstonei* from different hosts or geographic origins were downloaded from NCBI (for sequence information, see Additional file 1: Table S1) and aligned using ClustalW (https://www.genome.jp/tools-bin/clustalw). Maximum-likelihood and neighbour-joining models were used to construct analysis trees on the basis of the 18S rRNA, HSP70 and gGAPDH genes via MEGA 11, and bootstrap support with 1000 replicates was performed [23]. In addition, MEGA 11 was used to calculate the divergence time of the isolated *Trypanosoma* sp. from *T*. *cruzi* and *T*. *brucei* using the 18S rRNA sequences [23].

#### Infection experiment

To identify whether *Tr*. *rubrofasciata* could transmit trypanosomes, fresh triatomine faeces were mixed with one drop of PBS to detect trypanosomes directly via microscopy. Next, Kunming mice, C57BL/6J, BALB/cA-nu and Sprague‒Dawley rats were infected with these trypanosomes by intraperitoneal injection of the faeces. Blood samples were taken from the tails of the rats and mice every 12 h post infection to detect trypanosomes. Then, the infected mice and rats were bitten, and blood was collected from the laboratory-bred, uninfected *Tr*. *rubrofasciata*. After feeding, the fresh faecal matter of these bugs was checked to detect trypanosome infection using the method described above.

#### Transovarian transmission experiment

In total, six infected female *Tr*. *rubrofasciata* were used in this study. The laid eggs were collected in a container for hatching, and the first-stage nymphs were fed the Kunming mouse blood throughout their nymph life cycle and into the adult stage. The suspension drop method was used to detect trypanosomes in fresh faeces twice a week as described above.

#### Investigation of natural *Trypanosoma* sp. infection

To investigate the prevalence of trypanosomes in wild *Tr*. *rubrofasciata* populations**,** 135 triatomines, including 61 adults and 74 nymphs were collected. DNA was extracted from the abdominal tissues of the *Tr*. *rubrofasciata*, and a nested PCR strategy for detecting and identifying Trypanosoma species as previously described by Noyes et al. was used [24]. The PCR primer information is listed in Table 1, and the PCR amplification conditions were as follows: 94 °C for 3 min; 30 cycles of 94 °C for 30 s, 55 °C for 60 s, and 72 °C for 90 s; and 72 °C for 10 min. In addition, 16 *Rattus norvegicus* were collected near the triatomine collection sites in Chongzuo City. Blood samples were collected from the tail, and the blood was examined via trypanosomes. DNA was also extracted from the blood samples, and the DNA was amplified using the nested PCR method as described above to detect trypanosome infection [24]. All the positive PCR products were sequenced and subjected to BLAST to identify the Trypanosoma species.

## Results

### Triatomine specimen identification

All the triatomines were identified morphologically as *Tr*. *rubrofasciata*. The 16S rRNA and cytb PCR results of *T*. *rubrofasciata*, which yielded 499 bp and 667 bp fragments, respectively, were all identical and 100% matched those of the *Tr*. *rubrofasciata* in GenBank (accession nos. MH236905 and MH368021). The species was identified molecularly as *Tr*. *rubrofasciata*.

### Microscopic observations

**Fig. 1:**
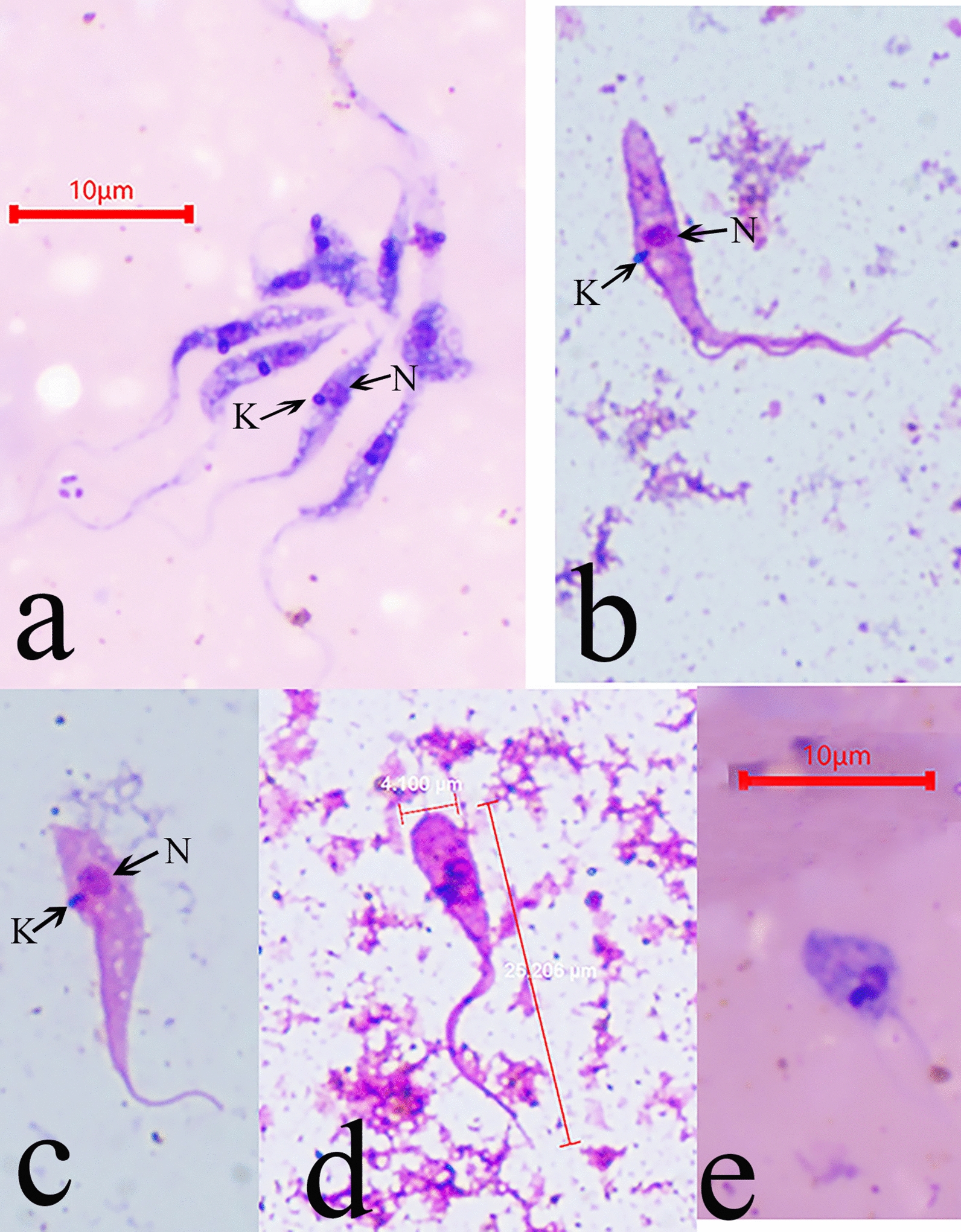
The morphology of *Trypanosoma* sp. in *Triatoma rubrofasciata*. **a** slender epimastigote form; **b** and **c** stumpy epimastigote form; **d** epimastigote form with round posterior end; **e** promastigote form

Flagellates were found in the faeces of the *Tr*. *rubrofasciata*. They were observed to have characteristics of *Trypanosoma* species and were motile (Additional file 1: Video S1). The faeces and intestinal contents were stained with Giemsa, and the results showed that the parasites mostly appeared as classical epimastigotes (Fig. 1a–d), but there were also a few promastigotes (Fig. 1e). According to morphology, the epimastigotes can be divided into three primary forms: the first is a slender epimastigote with a fine posterior end (Fig. 1a), the second is a stumpy epimastigote with a finger shape or fine posterior end (Fig. 1b, c) and the third is a rare epimastigote with a round posterior end (Fig. 1d).

**Fig. 2:**
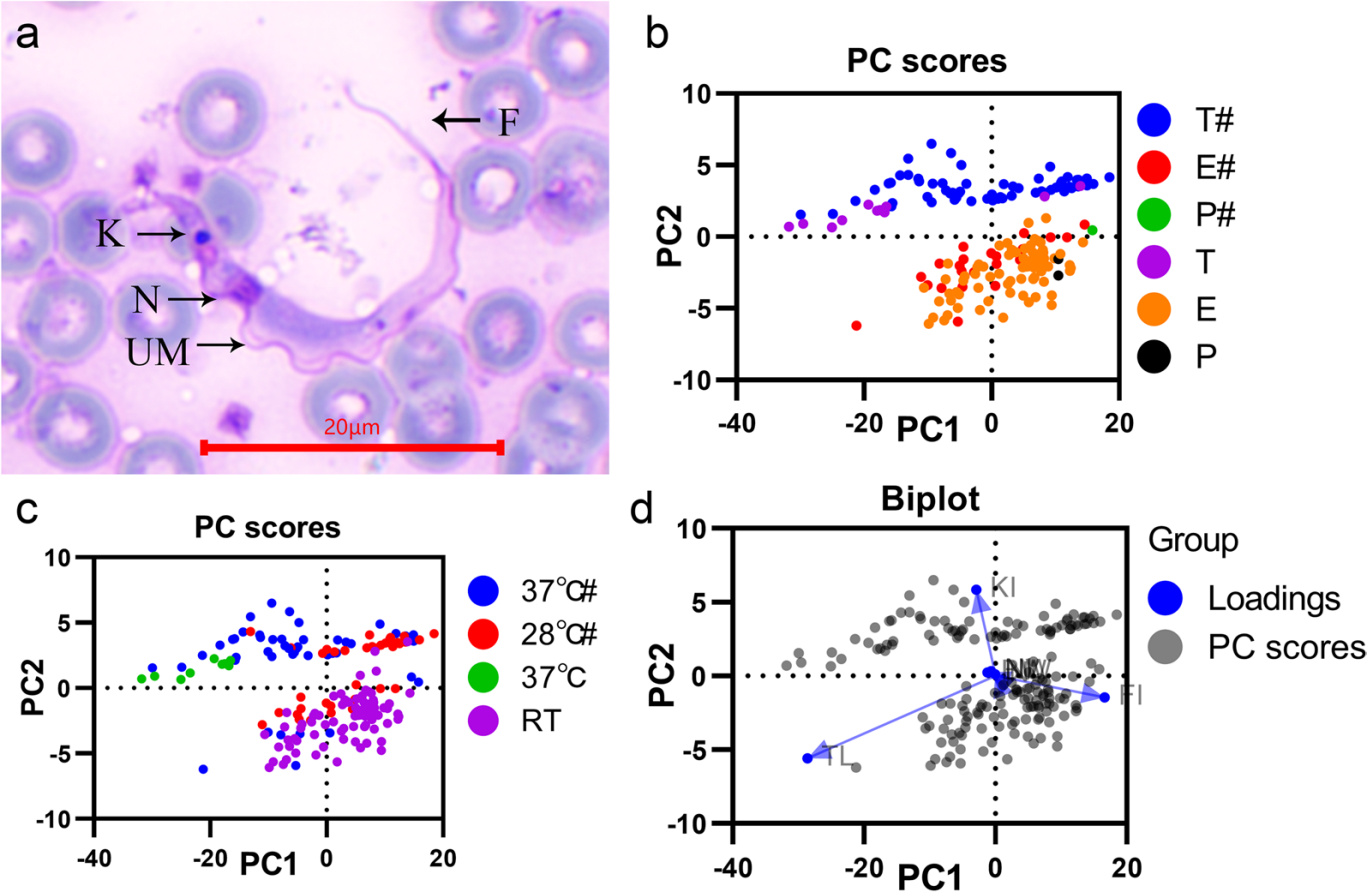
Morphological comparison of the *Trypanosoma* sp. (from rat and *Tr*. *rubrofasciata*) with *T*. *conorhini* in literature. **a** *Trypanosoma* sp. In lab infected rat; **b**–**d** Principal component analysis on morphological data (Table 2; TL: total cell length; BW: body width; NL, nucleus length; NW: nucleus width; NI: nucleus index, equals to PN/NA; KI: kinetoplast index, equal to PN/KN; and FI: flagellar index, equal to FF/BL) of our isolated trypanosomes with literature data. Grouping by cell forms (**b**) or by living temperature (**c**). The loading of parameters were indicated in blue (**d**). # data from T. conorhini drawings (Deane and Deane 13)

The nucleus, undulating membrane, flagellum and kinetoplast were apparent. The nucleus and the kinetoplast were close, with the former oval and anterior and the latter appearing as a dense dot far from the posterior extremity. The body length of the observed epimastigotes ranged from 6.3 to 33.9 µm, and the length of the free flagellum ranged from 2.3 to 19.0 µm (Table 2). After Giemsa staining, mice and rats infected in the laboratory were found to have typical trypomastigotes with well-developed undulating membrane bodies and clear flagellar pockets. The body length of the trypomastigotes was 36.7–52.5 µm, and the free flagellum length ranged from 4.8–10.5 µm (Fig. 2a, Table 2).

**Table 2:** Morphometrics of isolated trypanosomes compared with *T*. *conorhini*

Cell form^a^		KN^b^	PK	PN	NA	BW	BL	FF
E	RT	−1.4 ± 0.4	6.1 ± 1.9	5.1 ± 1.7	10.2 ± 5.6	2.1 ± 0.7	15.5 ± 6.7	8.9 ± 4.3
		−2.7 to −0.6	2.2–10.1	2.6–9.1	3.4–25.3	1.1–4.0	6.3–33.9	2.3–19.0
E	28 °C^c^	−3.4 to −1.5	4.6–9.7	3.6–10.0	5.0–17.7	1.0–4.1	8.5–24.6	2.1–15.7
E	37 °C^c^	−2.4 to −1.1	2.9–14.3	2.5–14.0	4.7–22.7	2.0–5.0	7.3–36.8	4.9–13.5
P	RT	−1.0	4.4–5.4	3.6–4.5	3.4–6.2	1.3–2.2	6.9–11.0	5.8–9.7
P	37 °C^c^	−1.4	4.5	3.0	4.9	3.0	7.8	3.2
T	RT	2.0–3.5	0.3–0.5	2.4–3.8	6.8–9.6	1.1–1.7	9.9–13.8	2.8–4.5
T	28 °C^c^	1.7–5.0	0.4–11.3	2.6–13.9	2.3–12.4	1.6–4.0	5.8–24.7	1.7–14.7
T	37 °C	5.4 ± 0.8	10.0 ± 1.3	15.0 ± 1.7	26.7 ± 3.5	2.6 ± 0.4	42.0 ± 5.0	6.5 ± 1.6
		4.4–7.0	7.9–12.2	12.7–17.9	22.9–34.5	1.9–3.2	36.7–52.5	4.8–10.5
T	37 °C^c^	1.6–6.5	0.4–16.7	1.7–23.0	2.4–24.7	1.6–4.9	6.9–42.3	1.2–15.1

Cell form^a^		TL	NL	NW	FL	NI	KI^b^	FI	*n*
E	RT	23.7 ± 6.8	1.9 ± 0.5	1.2 ± 0.3	18.5 ± 5.3	0.6 ± 0.4	− 4.6 ± 1.3	0.7 ± 0.5	81
		6.3–37.7	1.2–3.8	0.7–2.5	8.7–29.8	0.2–1.6	−7.6 to −1.4	0.1–1.9	
E	28 °C^c^	15.1–38.2	1.6–2.7	1.1–2.5	10.1–32.2	0.2–0.7	−4.1 to −2.2	0.1–0.8	17
E	37 °C^c^	12.2–48.5	2.0–2.6	1.2–2.5	8.9–50.0	0.4–1.1	−7.3 to −2.5	0.3–0.7	8
P	RT	16.6–16.7	1.2–1.7	1.1–1.3	11.1–13.2	0.7–1.1	−5.6 to −4.4	0.5–1.4	2
P	37 °C^c^	11.0	2.0	1.8	6.7	0.6	−3.1	0.4	1
T	RT	12.7–18.3	2.8–2.9	0.8–0.9	12.5–19.2	0.4–0.4	0.1–0.2	0.3–0.3	2
T	28 °C^c^	8.0–39.4	1.5–2.9	0.8–2.4	9.8–32.0	0.3–1.6	0.1–3.8	0.3–1.1	23
T	37 °C	48.6 ± 5.8	2.7 ± 0.5	1.9 ± 0.3	47.8 ± 5.5	0.6 ± 0.1	1.9 ± 0.3	0.2 ± 0.0	10
		43.0–58.4	2.1–3.6	1.6–2.4	41.1–58.7	0.5–0.7	1.4–2.4	0.1–0.2	
T	37 °C^c^	11.6–56.4	1.6–2.5	0.6–2.5	12.6–50.9	0.2–1.5	0.2–5.6	0.1–0.9	40

Biometric data (centre to centre distances across the cell axis) in μm are provided as mean ± SD and ranges

*KN* kinetoplast to nucleus, *PK* posterior end to kinetoplast, *PN* posterior end to nucleus, *NA* nucleus to anterior end, *BL* body length, *FF* free flagellum, *L* total length, *NL* nucleus length, *NW* nucleus width, *BW* body width, *NI* nucleus index (PN/NA), *KI* kinetoplast index (PN/KN), *FI* flagellum index (FF/BL), *RT* room temperature

^a^E for epimastigote, P for promastigote and T for trypomastigotes

^b^Minus value is applied for KN and KI values of epimastigotes and promastigote, as kinetoplast position is prior to nucleus position

^c^Measurement of *T*. *conorhini* drawings from Deane and Deane [13], therefore mean and SD are not calculated to avoid bias interpretation

A comparison of the morphological characteristics of the isolated trypanosomes and *T*. *conorhini* from literature [13] is shown in Table 2 and Fig. 2b–d based on measurements of the distance and the index below. As expected, the morphological data were split into two major groups with regard to the kinetoplast index (KI, equal to PN/KN), trypomastigotes and the rest (epimastigotes and promastigotes). The distribution of these two major groups, which stretched nearly horizontally into stratiform regions, was attributed to the total length of the cell (TL, equal to BL plus FF) and the flagellar index (FI, equal to FF/BL). A comparison of these data for the isolated *Trypanosoma* sp. with specific *T*. *conorhini* data from the literature strongly suggested a close relationship.

### Phylogenies based on the 18S rRNA, gGAPDH and HSP70 sequences

After cloning and sequencing, we obtained 2018 bp, 922 bp and 769 bp fragments of the 18S rRNA, gGAPDH (accession no. MZ043866) and HSP70 (accession no. MZ043865) sequences, respectively. BLAST analysis revealed that the closest 18S rRNA sequence was most similar (99.7%) to that of the *T*. *conorhini* strain Tco025E (accession no. MKKU01000460), which was isolated from *R*. *rattus* in Brazil. BLAST analysis of gGAPDH fit with *T*. *conorhini* Tco025E_10018 (99.0%, accession no. XM_029376810). HSP70 fit with *T*. *conorhini* Tco025E_09744 (99.0%, accession no. XM_029376553), which was isolated from *R*. *rattus* in Brazil, and TCC2156 (accession no. MF144909), which was isolated from *Tr*. *rubrofasciata* in the USA.

**Fig. 3:**
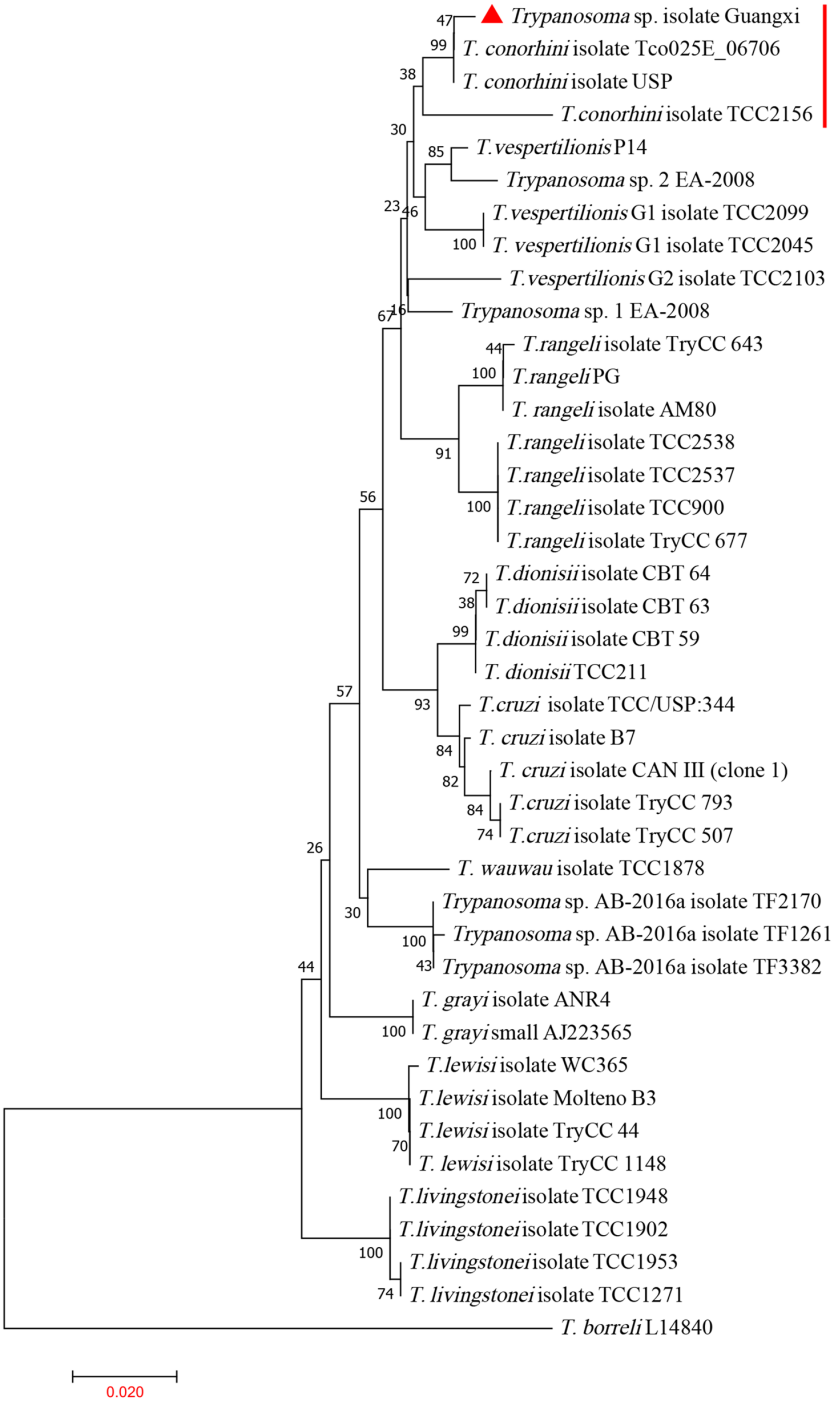
The phylogenetic tree is based on the 18S rRNA gene sequences from newly isolated *Trypanosoma* sp. and other related species. The phylogenetic tree was constructed by MEGA using the neighbour-joining (NJ) method with 1000 bootstrap replications. The accession numbers of 18S rRNA sequences of *Trypanosoma* spp. in GenBank are in Additional file 1: Table S1. The phylogenetic analysis showed the isolated *Trypanosoma* sp. is close with *T*. *conorhini*

**Fig. 4:**
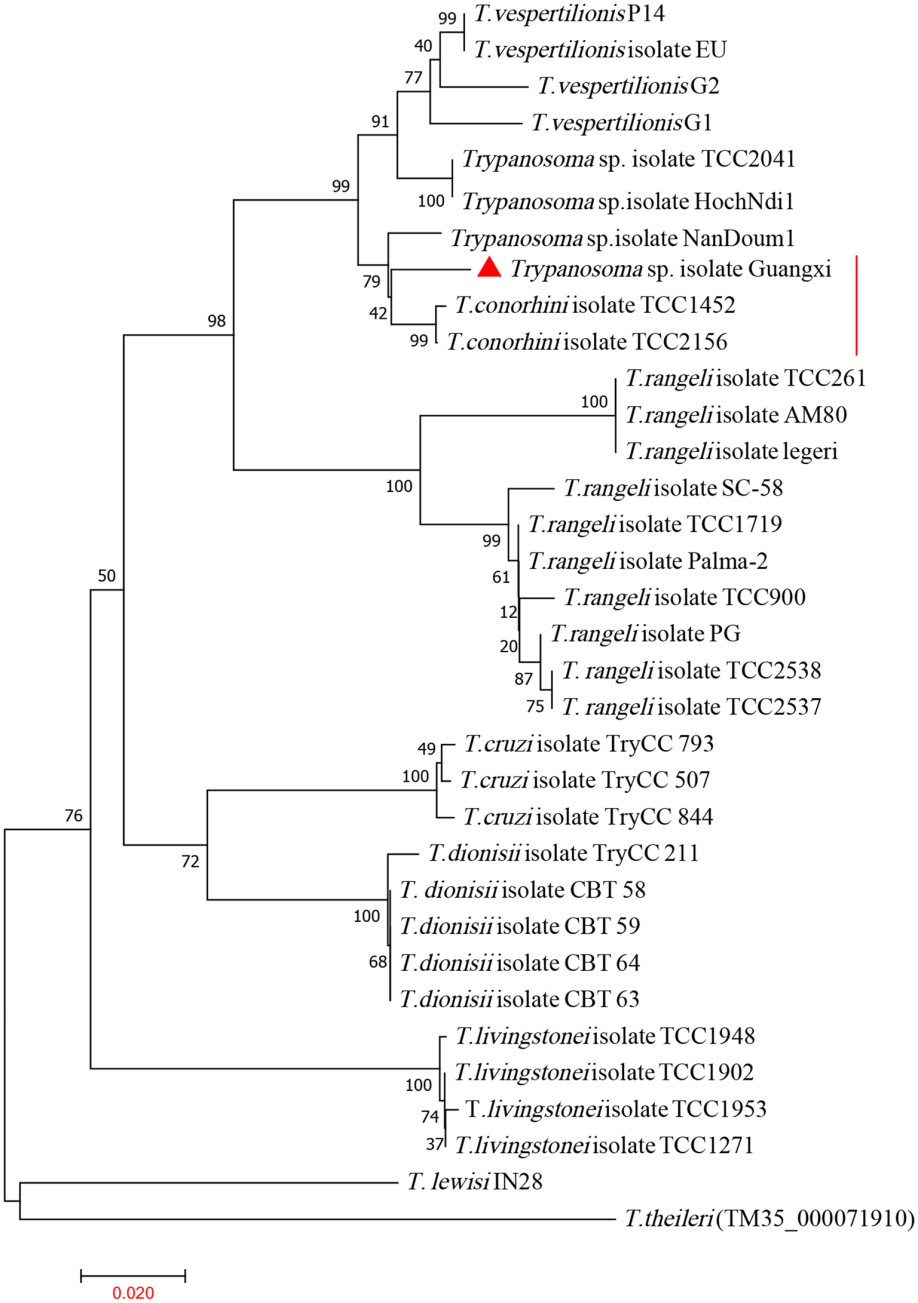
The phylogenetic tree is based on the heat shock protein 70 sequences from newly isolated *Trypanosoma* sp. and other related species. The phylogenetic tree was constructed by MEGA using the neighbour-joining (NJ) method with 1000 bootstrap replications. The accession numbers of gGAPDH sequences of *Trypanosoma* spp. in GenBank are in Additional file 1: Table S1. The phylogenetic analysis showed the isolated *Trypanosoma* sp. is close with *T*. *conorhini*

**Fig. 5:**
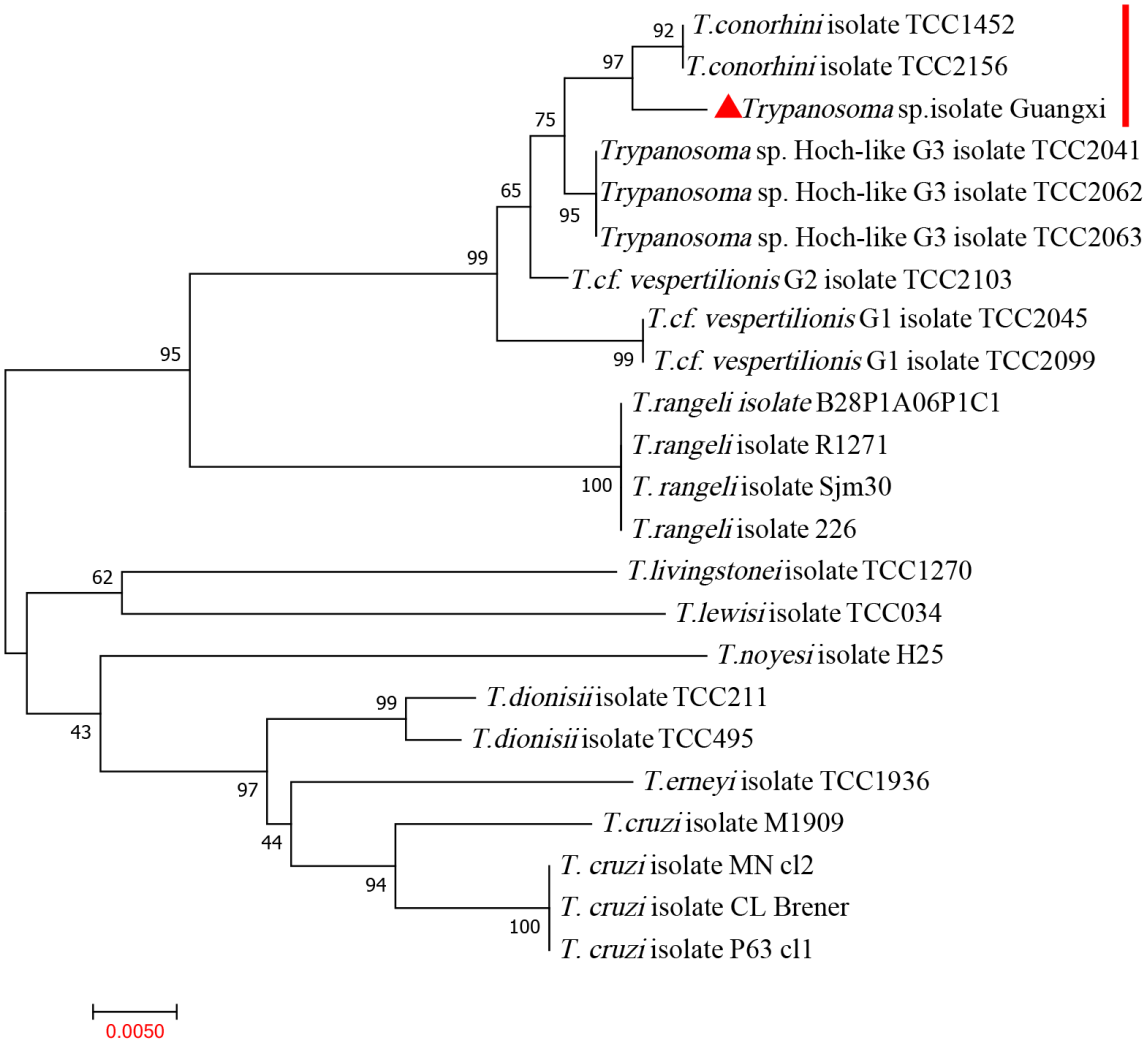
The phylogenetic tree is based on the glycosomal glyceraldehyde-3-phosphate dehydrogenase sequences from newly isolated *Trypanosoma* sp. and other related species. The phylogenetic tree was constructed by MEGA using the neighbour-joining (NJ) method with 1000 bootstrap replications. The accession numbers of HSP70 sequences of *Trypanosoma* spp. in GenBank are in Additional file 1: Table S1. The phylogenetic analysis showed the isolated *Trypanosoma* sp. is close with *T*. *conorhini*

The obtained 18S rRNA, HSP70 and gGAPDH sequences were subjected to phylogenetic tree construction with representative species of all major trypanosome clades (Figs. 3, 4, 5). The new triatomine trypanosomes formed a well-supported clade close to *T*. *conorhini* and *T*. *vespertilionis* in all phylogenetic trees. According to the 18S rRNA phylogenetic trees, the newly isolated cluster contained four *T*. *conorhini* isolates from the *Tr*. *rubrofasciata* and rats: TCC1452, TCC2156, USP and Tco025E; and three *T*. *verspertilionis* isolates from bats and bugs: G1, G2, and P14. The newly isolated cluster was distinct from those of *T*. *rangeli* and *T*. *cruzi* (Fig. 3). In the gGAPDH and HSP70 phylogenetic trees, the newly isolated clade with two isolates of *T*. *conorhini* (TCC1452 and TCC2156) was found to be closer to the *Trypanosoma* sp. Hoch-like G3 and HochNdil (from bats and monkeys) and *T*. *vespertilionis* G1, G2, EU and P14 clades than to the *T*. *rangeli* and *T*. *cruzi* clades (Figs. 4, 5).

**Fig. 6:**
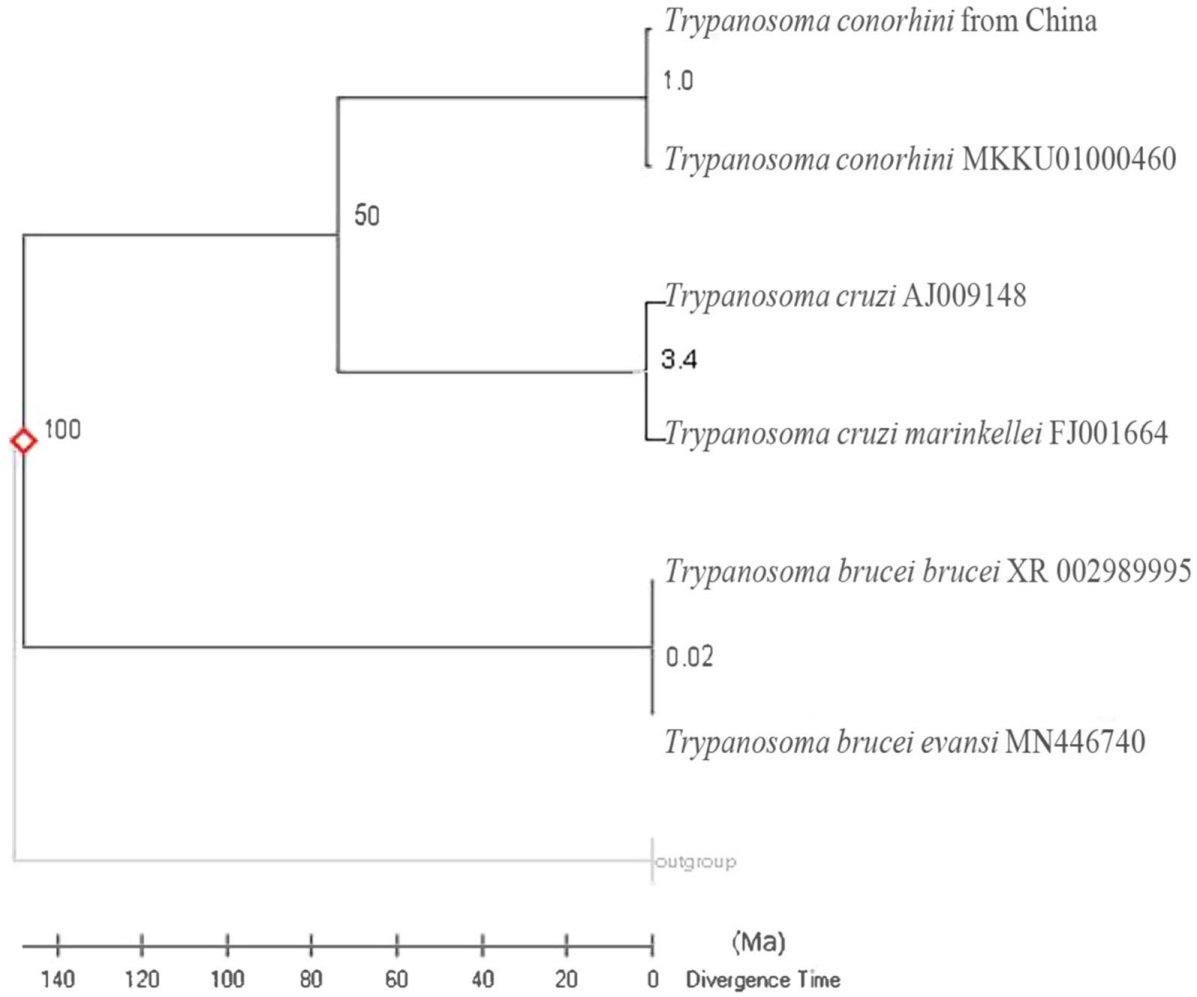
Divergence time calculation of the isolated Chinese *T*. *conorhini* and South American strains

Additional sequencing of ITS1 (652 bp) from the isolated trypanosomes revealed 98.8% similarity to the *T*. *conorhini* reference strain Tco025E (accession no. MKKU01000460). Overall, this newly isolated trypanosome genome is the first published record of a Chinese *T*. *conorhini*. By applying a possible divergence time of 100 million years ago (MYA) between *T*. *cruzi* and *T*. *brucei brucei* [25], we estimated a possible divergence time of 1 Ma between Chinese *T*. *conorhini* and the South American strains (Fig. 6).

### *Trypanosoma* sp. experimental transmission

Experimental infection of the Chinese *T*. *conorhini* showed that the trypanosomes were detected 2 or 3 days after inoculated faeces containing trypanosome were injected through intraperitoneal injection (Additional file 1: Video S2). The Kunming mice and C57BL/6J, BALB/C, BALB/cA-nude and Sprague‒Dawley rats developed low parasitaemia, and the parasitaemia (fewer than 1 × 10^2^ parasites per ml) persisted for more than 1 month*.* The infected rats and mice did not display symptoms, such as anaemia or depression. Trypanosomes were detected in the *Tr*. *rubrofasciata* faeces at 1 week after feeding on infected mice through optical microscopic observation.

To test the potential for transovarian transmission, experiments were conducted for 20 weeks until all the nymphs of the next generation *Tr*. *rubrofasciata* became adults. No trypanosomes were detected in any of the fresh *Tr*. *rubrofasciata* faeces throughout the experiment.

### Investigation of natural *Trypanosoma* sp. infection

Overall, 49 of the 135 triatomine specimens collected in Guangxi were PCR positive for approximately 600 bp fragments, with an infection rate of 36.30%. The adult and nymph infection rates were 39.3% (24/61) and 33.8% (25/74), respectively. The sequences of the PCR products revealed 98.1–99.7% similarity to the *T*. *conorhini* reference strain Tco025E (accession no. MKKU01000460). However, trypanosomes were neither observed or detected by PCR in the blood smears from 16 *R*. *norvegicus*.

## Discussion

In this study, we identified *T*. *conorhini* isolates from the triatomine *Tr*. *rubrofasciata* in China for the first time. The trypanosome species were confirmed by both morphological and molecular methods. Trypanosome species identification by morphological methods is challenging because molecular methods improve the accuracy of identification. Phylogeny based on the 18S rRNA, HSP70 and gGAPDH genes has been used for evolutionary and taxonomic studies of trypanosomatids, and it is recommended that all new trypanosome species be phylogenetically validated using at least two of these genes [26–29]. In this case, we employed three genes to confirm this species; with the obtained phylogenetic trees showing that the new Chinese triatomine trypanosomes belong to the *T*. *conorhini* clade with limited differences compared with the species documented from the USA and Brazil [30, 31]. This finding is convincing, as the genetic identity is based on both morphological observations and molecular analysis*.*

*Trypanosoma conorhini* is one of four trypanosomes (*T*. *cruzi*, *T*. *rangeli*, *T*. *conorhini* and *T*. *lewisi*) that have been reported to be transmitted by *Tr*. *rubrofasciata* via blood feeding [15]. Therefore, *T*. *conorhini* deserves more attention for the following reasons: (1) *T*. *conorhini* is a close relative of known human pathogens. The other three trypanosomes found in the *Tr*. *rubrofasciata* are closely related to *T*. *conorhini* and known human pathogens. *Trypanosoma cruzi* and *T*. *rangeli* cause human infection, and *T*. *lewisi* (also isolated from *Tr*. *rubrofasciata*) can cause atypical human infection [32]. The study of *T*. *conorhini* revealed that there are human pathogens in addition to *T*. *cruzi*, suggesting that *T*. *conorhini* should also be classified as a neglected human pathogen even if it is thought to be nonpathogenic to humans and is generally transmitted to a restricted nonhuman host range, where it causes mild and transient infection. For example, laboratory experiments have shown that nonhuman primates can become infected with *T*. *conorhini* [12, 13]. More importantly, cutaneous symptoms in humans after bites by *Tr*. *rubrofasciata* are common [16]. (2) Population growth of *Tr*. *rubrofasciata* increases the possibility of human encounters with *T*. *conorhini*. After all, *Tr*. *rubrofasciata* is the only globally distributed triatomine, and its prevalence has been reported to increase significantly in several Asian countries [33, 34]. Moreover, previous investigations have shown a rising number of reports of *Tr*. *rubrofasciata* bites in humans across regions of China, which is becoming a public health problem because it can also cause severe anaphylactic reactions [17, 20].

In our study, Chinese *T*. *conorhini* was not transovarially transmitted in the *Tr*. *rubrofasciata*. However, the infection ratio of *T*. *conorhini* in the *Tr*. *rubrofasciata* reached 36.30%. The prevalence of *T*. *conorhini* infection in triatomines in China is very similar to that reported in Vietnam [15]. However, the natural host of this parasite has yet to be identified, and larger-scale investigations are needed. Our results demonstrated that rats and mice could be infected under laboratory conditions with *T*. *conorhini* via incubated faeces or bites from the *Tr*. *rubrofasciata* collected from the wild. In turn, the *Tr*. *rubrofasciata* also became infected from ingesting infected rats and mouse blood. This indicated that *Tr*. *rubrofasciata* is a vector for *T*. *conorhini*. Furthermore, this is supported by a paper describing a high number of *Tr*. *rubrofasciata* found near chicken coops [20]. Therefore, future work should investigate the possibility of chickens as reservoir hosts.

Finally, we used molecular clock analysis and predicted a divergence time of 1 MYA between Chinese and South American *T*. *conorhini*. This corresponds to the time when hominids dominated Asia, i.e. *Homo erectus* arrived in the southeastern part of Asia at approximately 1.7 MYA [35, 36]. However, it is unclear whether the prevalence of *Tr*. *rubrofasciata* and *T*. *conorhini* in Asia can be associated with the hominids of this time period or with other mammals or birds.

In addition to *T*. *conorhini*, many new trypanosome species have recently been reported in China, e.g. *T*. *evansi* in cattle and buffalo [37]; *T*. *dionisii*, *T*. *wauwau* and other *Trypanosoma* sp. in bats [38, 39]; *T*. *lewisi* from rats and mice [40]; *T*. *carassii* in fish [41]; and *Trypanosoma* (*Megatrypanum*) *bubalisi* sp. *nov*. in leeches [42]. This finding for *T*. *conorhini* in China reflects how limited our knowledge of trypanosomes and vectors is.

## Conclusions

In this study, a *Trypanosoma* sp. was found in *Tr*. *rubrofasciata* in China, the morphology and molecular analysis of which indicated that this trypanosome was *T*. *conorhini*. However, we ruled out that transovarial transmission by *Tr*. *rubrofasciata* of this Chinese *T*. *conorhini* is not possible; it can be transmitted between *Tr*. *rubrofasciata* and laboratory mice and rats. There was a higher prevalence (> 36%) of *T*. *conorhini* in the *Tr*. *rubrofasciata* collected from our collection sites than from mice and rats collected from other sites. Future work should investigate whether *T*. *conorhini* is pathogenic to humans.

### Supplementary Information

**Additional file 1: Video S1.** The moving of *Trypanosoma* sp. in *T. rubrofasciata* faeces from Guangxi. **Video S2.** The moving of *Trypanosoma* sp. in blood of infected mice. **Table S1.** The trypanosomes sequences information that downloaded from NCBI and used in the phylogenetic analysis.

## Data Availability

The data supporting the finds of the study are available within the article and its supplementary materials and genbank.

## Abbreviations

NJ: Neighbour-joining
HSP70: Heat shock protein 70
gGAPDH: Glycosomal glyceraldehyde-3-phosphate dehydrogenase
ITS: Internal transcribed spacer region
